# Performance of Dried Blood Spot Samples in SARS-CoV-2 Serolomics

**DOI:** 10.3390/microorganisms10071311

**Published:** 2022-06-29

**Authors:** Rima Jeske, Uta Merle, Barbara Müller, Tim Waterboer, Julia Butt

**Affiliations:** 1 Division of Infections and Cancer Epidemiology, German Cancer Research Center, 69120 Heidelberg, Germany; t.waterboer@dkfz-heidelberg.de; 2 Faculty of Biosciences, Heidelberg University, 69120 Heidelberg, Germany; 3 Department of Internal Medicine IV, University Hospital Heidelberg, 69120 Heidelberg, Germany; uta.merle@med.uni-heidelberg.de; 4 Department of Infectious Diseases, Virology, University Hospital Heidelberg, 69120 Heidelberg, Germany; barbara.mueller@med.uni-heidelberg.de

**Keywords:** SARS-CoV-2, COVID-19, dried blood spots, multiplex serology, serolomics

## Abstract

Numerous sero-epidemiological studies have been initiated to investigate the spread and dynamics of severe acute respiratory syndrome coronavirus 2 (SARS-CoV-2). To address the concomitant need for serological high-throughput assays, a bead-based multiplex serology assay, specific for SARS-CoV-2, had been developed. SARS-CoV-2 serolomics allows for measuring antibody responses to almost the entire SARS-CoV-2 proteome in up to 2000 serum samples per day. To enlarge the pool of eligible sample collection methods, we here test the compatibility of serolomics with dried blood spot (DBS)-derived eluates. Antibody levels of nine SARS-CoV-2 antigens, including the nucleocapsid (N) and receptor-binding domain of the spike protein (S1-RBD), were measured in 142 paired DBS and serum samples. The numeric correlation between the two sample types was high, with a Pearson’s r of 0.88 for both S1-RBD and N and intraclass correlation coefficients of 0.93 and 0.92, respectively. Systematically reduced antibody levels in DBS eluates were compensated by lowering the cutoffs for seropositivity accordingly. This enabled the concordant classification of SARS-CoV-2 seropositivity, without loss in sensitivity. Antibody levels against accessory SARS-CoV-2 antigens also showed a high concordance, demonstrating that DBS-derived eluates are eligible for SARS-CoV-2 serolomics. DBS cards facilitate the collection of blood samples, as they obviate the need for medically trained personnel and can be shipped at room temperature. In combination with SARS-CoV-2 serolomics, DBS cards enable powerful sero-epidemiological studies, thus allowing for the monitoring of patients and epidemiological analyses in resource-poor settings.

## 1. Introduction

With the outbreak of severe acute respiratory syndrome coronavirus 2 (SARS-CoV-2) in 2019, many sero-epidemiological studies have been initiated [1,2]. In combination with molecular diagnostic testing, screening blood samples for antibodies against SARS-CoV-2 can reveal the total burden of exposure in the general population and guide public health policy [3]. The collected data can further be employed for various analyses, e.g., for modeling longitudinal spread and dynamics of the pandemic, or retrospective evaluation of the efficacy of vaccination strategies [4,5].

Addressing the concomitant need for cost-effective serological high-throughput assays, we recently developed SARS-CoV-2 serolomics, a bead-based multiplex serology assay incorporating almost the entire SARS-CoV-2 proteome, including the nucleocapsid (N), receptor-binding domain of the N-terminal subdomain of the spike protein (S1-RBD), and other structural, non-structural, and accessory proteins [6]. With multiplex serology, relative antibody levels against up to 100 different antigens can be simultaneously measured in up to 2000 samples per day, while requiring only a small volume of serum. Prior exposure to SARS-CoV-2 can be assessed by measuring the antibody levels against S1-RBD and N. Seropositivity to other SARS-CoV-2 antigens is less common but might be of clinical relevance, due to an association with disease progression or transmission, as currently debated [7,8,9]. Multiplex serology further enables studying SARS-CoV-2 in the context of other infectious pathogens. The assay platform currently incorporates autoantigens and antigens from diverse microorganisms, including different herpes viruses, human papilloma viruses, *Chlamydia trachomatis*, and *Helicobacter pylori* [10,11,12,13,14,15]. The repertoire can easily be expanded, e.g., by antigens from other respiratory pathogens.

As serological assays in general, multiplex serology depends on the availability of suitable serum or plasma samples. Although blood draws are performed routinely, they still require medically trained staff for the intravenous puncture, as well as equipment and infrastructure to store and ship samples below 0 °C.

Dried blood spot (DBS) cards pose a practical alternative for collecting sample materials [16,17]. Drops of full blood, e.g., from the fingertip or earlobe, are applied on these filter papers and dried. Sample preparation can be performed without medical training, or even self-administered, which obviates the need for study participants to visit a study center. This is particularly relevant if infected persons need to quarantine and also facilitates obtaining multiple consecutive samples for longitudinal studies [18,19]. Additionally, sampling intervals can be modified, e.g., to gain longitudinal samples capturing the kinetics of different SARS-CoV-2 antibodies [19,20].

Furthermore, shipping and storing serum samples below 0 °C can pose a major logistic challenge, especially in resource-poor settings that had led to an underrepresentation among sero-epidemiological SARS-CoV-2 studies [1]. This problem is also addressed by DBS cards, which can be shipped and stored at room temperature for a prolonged time [21].

Overall, the use of DBS samples could substantially aid the surveillance of SARS-CoV-2 and analyzing of longitudinal serological studies [18,19].

In this proof-of-principle study, we tested the compatibility of DBS samples and multiplex serology by measuring the antibody levels to nine SARS-CoV-2 antigens in DBS eluates and paired serum samples. Numerical readouts were compared, in order to evaluate whether DBSs are a suitable substitution for conventional serum samples and eligible for SARS-CoV-2 serolomics. 

## 2. Materials and Methods

### 2.1. Study Population

The cohort analyzed in this proof-of-principle analysis was previously characterized in detail, by Seeßle et al. [22]. Briefly, SARS-CoV-2 patients, formerly treated as out- or inpatients at the Department of Internal Medicine IV of the University Hospital Heidelberg, were invited into a prospective study focusing on the long-term effects after acute infection (Ethics Committee of University of Heidelberg, reference number: S-546/2020; DRKS00025089). Blood samples were obtained at baseline (10–18 weeks after symptom onset), after 5 months (20–22 weeks), after 9 months (33–40 weeks), and after 12 months (50–54 weeks), in compliance with health and safety rules (gloves, face masks, and safety glasses).

For the here presented DBS validation paired serum samples and DBS cards from 142 patients, collected at the first follow-up visit (5 months after symptom onset) were available. 

### 2.2. Sample Preparation and Processing of DBS Cards

Each study participant had 18 mL full blood drawn by venipuncture, of which 70 µL were directly applied to DBS cards (Whatman 903 protein saver blood collection cards;Whatman, Maidstone, UK ) using a syringe. The remaining blood was centrifuged to isolate the serum and stored at −20 °C. DBS cards were shipped to the German Cancer Research Center (DKFZ), together with the corresponding sera, at room temperature and −20 °C, respectively. DBS cards were stored at 4 °C for approximately nine months prior to usage. To elute antibodies from DBS cards, a punch of 6 mm diameter was prepared and agitated in 180 µL PBS at 4 °C overnight. Assuming complete elution of the material, the DBS eluate corresponds to 15.8 µL full blood or 8.7 µL serum, as described by Waterboer et al. [23].

### 2.3. Multiplex Serology and Antigen Selection

The expression of SARS-CoV-2 antigens and the experimental procedure were previously described in detail [6,14]. For the here presented study, we selected the best performing structural antigens, i.e. N and S1-RBD. Furthermore, seven non-structural proteins (NSP) and accessory antigens (open reading frame; ORF) (ORF3a, ORF9, NSP2, NSP7, NSP8, NSP10, and NSP15) were selected as representatives for the less seroprevalent antigens. As non-SARS-CoV-2 controls, antigens from three endemic human viruses were included: the major capsid proteins (VP1) from the BK polyomavirus (BK) and JC polyomavirus (JC) and envelope glycoproteins gE/gI (co-loaded) from varicella-zoster virus (VZV).

Briefly, all antigens, except for S1-RBD, were expressed as GST-X-tag fusion proteins, affinity-purified on spectrally distinguishable glutathione–casein-coupled beads (SeroMap, Luminex Corp., Austin, TX, USA) and combined into a single bead mix. S1-RBD was recombinantly expressed as His-tagged protein in HEK-293 cells, as described by Butt et al., and directly cross-linked to the beads surface [6]. To suppress unspecific binding, serum samples were incubated in a 1:50 dilution in a preincubation buffer containing 0.5% polyvinyl alcohol, 0.8% polyvinyl pyrrolidone, casein, 2 mg/mL *E. coli* GST-tag lysate, and 2.5% Super ChemiBlock™ heterophile blocking agent (CBS-K, Chemicon, Temecula, CA, USA). For preincubation of the DBS eluates, 32 µL were combined with 102 µL of preincubation buffer, corresponding to a similar dilution of 1:66 for the calculated serum content. After the preincubation step, both sample types were each combined with the same volume of bead mix to a final serum dilution of 1:100 and 1:133, respectively. Subsequently, a biotinylated secondary anti-human IgG/IgA/IgM antibody (1:1000, #109-065-064, Lot: 149169, Jackson Immunoresearch, West Grove, PA, USA) and streptavidin-R-phycoerythrin (1:750, MossBio, Pasadena, MD, USA) were used to detect bound serum antibodies. Signal quantification and identification of the respective bead type were conducted using a Luminex 200 instrument (Luminex Corp., Austin, TX, USA). Readouts were given as median fluorescent intensity (MFI). 

### 2.4. Data Processing and Statistical Analysis

Raw MFI values were corrected by subtraction of the blank value, as determined in sample-free negative controls and the sample-specific GST-background. Numerical values were descriptively analyzed by medians and interquartile ranges (IQR). MFI values derived from paired serum and DBS samples were visualized as scatter plots, and linear regression was applied to show the general trend between the two sample types. Additionally, Bland–Altman plots were prepared to visualize trends across antibody level ranges. Correlation between the paired samples was calculated using Pearson’s r and interpreted as follows: 0.0–0.3: negligible; 0.3–0.5: low; 0.5–0.7: moderate; 0.7–0.9: high; 0.9–1.0: very high. The reliability between the two sample types was further described by the intraclass correlation coefficient (ICC) and interpreted as follows: <0.5: low; 0.5–0.75: moderate; 0.75–0.9: high; >0.9: very high. All statistical tests were performed with R (version 4.1.3). Plots were prepared using GraphPad prism 9.0.

## 3. Results

The antibody levels of nine SARS-CoV-2 antigens, as well as of three control antigens from endemic human viruses were determined in 142 paired DBS eluates and serum samples. Median MFI values and corresponding IQRs are specified in Table 1. 

**Figure 1:**
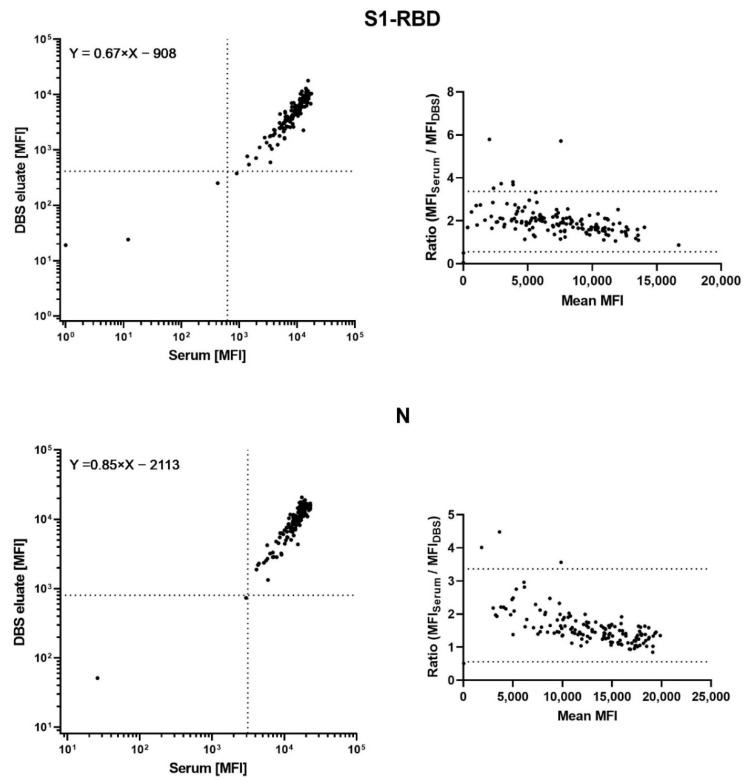
**Left**: Antibody responses to two structural SARS-CoV-2 antigens (S1-RBD and N), as measured in 142 paired serum and DBS samples and visualized as scatter plots. Linear regression was applied to estimate the general trend between the two data sets. Dotted lines represent respective cutoff values for seropositivity. **Right**: Bland–Altman plots visualizing the ratio between paired antibody responses across the MFI scale. Dotted lines are located at the mean +/−1.96 standard deviations of the ratios.

In serum samples, the structural antigens S1-RBD and N exhibited median antibody levels of 9497 MFI (IQR: 6212–12,618 MFI) and 15,743 MFI (IQR: 11,960–18,296 MFI), while readouts of the corresponding DBS eluates reached 5117 MFI (IQR: 2758–7585 MFI) and 10,867 MFI (IQR: 7042–14,368 MFI), respectively. The numerical correlation between paired samples was high, with a Pearson’s r of 0.88 for both antigens. ICCs were as high as 0.92 (95% CI: 0.88–0.94) and 0.93 (95% CI: 0.91–0.95), respectively. The absolute antibody levels determined in the DBS samples were, on average, reduced by the factors 1.5 and 1.2, respectively, as visualized by regression slopes of 0.67 and 0.85. As visualized by Bland–Altman plots, the relative reductions decreased with increasing MFI values (Figure 1).

The seroprevalences of S1-RBD and N were 98% and 99% in the serum samples. To achieve an ideal agreement, despite the lower antibody levels, cutoffs for seropositivity were reduced to 300 and 800 MFI for DBS eluates, respectively (Table 1 and Figure 1).

These observations were confirmed for the three control antigens, with a mean signal reduction by the factor 1.5 and regression slopes between 0.61 and 0.75 (Supplementary Figure S1). The correlation was concordantly high with Pearson’s r values between 0.90 and 0.96 and ICCs between 0.93 and 0.94.

The non-structural and accessory SARS-CoV-2 antigens ORF3a, ORF6, NSP2, NSP7, NSP8, NSP10, and NSP15 showed lower seroprevalences and MFI values than the structural proteins. Between 70% and 99% of the antibody level measurements fell below the lower limit of quantitation (100 MFI) or below the cutoff for seropositivity defined by Butt et al. [6]. The high portion of seronegative background noise led to decreased in ICC values between 0.51 (95%CI: 0.32–0.65) for NSP10 and 0.87 (95%CI: 0.82–0.91) for ORF3a.

Nevertheless, serum samples with high MFI values were also elevated in DBS eluates (Supplementary Figure S2). This is affirmed by a high numerical correlation, with a mean Pearson’s r of 0.88.

## 4. Discussion

In summary, we showed that a concordant classification of SARS-CoV-2 seropositivity can be achieved using DBS eluates in comparison to the corresponding serum samples.

Systematically reduced MFI values were compensated by lowering the cutoffs accordingly, as the results for the paired samples showed high numerical correlations. This reduction can be partially attributed to a higher final dilution of 1:133 for DBS eluates, while the serum samples were measured in a dilution of 1:100. Incomplete elution of antibodies from the filter paper and prolonged storage at 4 °C are further potential factors contributing to the diminished signals [21]. This is, however, a moderate systematic effect and not specific for SARS-CoV-2, as it applied to the control antigens to the same degree.

In theory, reduced antibody levels could be compensated by increasing the volume of DBS eluate in the assay. However, increasing the sample volume in relation to the preincubation buffer can impair the signal-to-noise ratio, which had been previously optimized for multiplex serology [23]. Adjusting the cutoffs for seropositivity is a feasible solution for maximizing the agreement between the two sample types.

As recently summarized by Amini et al. in a systematic review, antibody concentrations in DBS eluates depend on card types, storage conditions, and elution protocols [21,24]. As these factors will vary between different DBS-based sero-epidemiological studies, cutoffs need to be determined for the respective study, e.g. by finite mixture modeling [25]. Sample-type specific standards are desirable in order to enable a simple extrapolation of cutoffs. However, this approach only allows us to correct for the technical differences from assay to assay, but not for the differences in sample qualities that potentially result from the sampling methods, storage conditions, etc.

For the here presented study DBS cards were stored at 4 °C for nine months, which deviates from the optimal storage conditions below −20 °C [21,24]. Still, SARS-CoV-2 serolomics results were comparable to freshly thawed serum samples, which is promising for future DBS-based sero-epidemiological studies. Additional studies are necessary in order to estimate the variations between the different DBS preparations.

It needs to be further pointed out that the DBS cards for this study were prepared by trained medical personnel using intravenous blood, representing an optimized surrogate condition. Although the procedure principle of fingerprick blood collection and application is simple, it is well-known that the incorrect handling of DBS cards poses a major pitfall, especially if too little or too much blood is applied [17,19,26]. Therefore, detailed instructions need to be provided, and, if possible, adequate controls (e.g., control antigens from endemic pathogens with a high prevalence as polyomaviruses) should be included. Alternatively, quantitative DBS sampling cards could be used. These cards are supplemented with device inlet ports, thus ensuring the application of a pre-specified blood volume [26].

## 5. Conclusions

We showed that blood sampling by DBS cards in conjunction with multiplex serology represents a powerful tool that can facilitate the sero-epidemiological research of SARS-CoV-2. Potential applications include surveillance studies by mail or longitudinal sampling to study the kinetics of specific antibody responses [26]. Furthermore, low-resource countries could profit from the reduced logistical burden. Despite being particularly important for SARS-CoV-2 research, due to the low vaccination coverage globally, corresponding sero-epidemiological investigations are still underrepresented [1].

## Figures and Tables

**Table 1:** Antibody levels of nine SARS-CoV-2 antigens and three control antigens, as measured in 142 paired DBS and serum samples.

		Serum			DBS				
		Median Antibody Readout [MFI] (IQR)	Cutoff [MFI]	Seroprev. [%]	Median Antibody Readout [MFI] (IQR)	Cutoff [MFI]	Seroprev. [%]	Pearson’s r	ICC (3,k)
Structural antigens	**S1-RBD**	9497 (6212–12,618)	626	98	5117 (2758–7585)	300	98	0.88 (0.83–0.91)	0.92 (0.88–0.94
	**N**	15,743 (11,960–18,296)	3133	99	10,867 (7042–14,368)	800	99	0.88 (0.83–0.91)	0.93 (0.91–0.95)
Non- structural and accessory antigens	**ORF3a**	98 (46–374) *	287	30	54 (41–139) *	120	30	0.97 (0.96–0.98)	0.87 (0.82–0.91)
	**ORF6**	6 (1–12) *	100	3	28 (23–35) *	100 **	2	0.97 (0.96–0.98)	0.60 (0.44–0.71)
	**NSP2**	287 (166–801)	1391	17	203 (152–326)	430	17	0.92 (0.89–0.94)	0.78 (0.69–0.84)
	**NSP7**	1 (1–15) *	1719	2	8 (5–17) *	620	2	0.98 (0.98–0.99)	0.84 (0.78–0.89)
	**NSP8**	17 (6–32) *	849	1	10 (7–20) *	160	1	0.81 (0.74–0.86)	0.52 (0.33–0.66)
	**NSP10**	16 (10–29) *	147	7	16 (11–22) *	100 **	2	0.83 (0.77–0.87)	0.51 (0.32–0.65)
	**NSP15**	26 (18–40) *	135	4	19 (13–28) *	100 **	1	0.71 (0.61–0.78)	0.65 (0.51–0.75)
Control antigens	**BK**	13,533 (5312–16,303)	250	100	7540 (2398–11,161)	100 **	100	0.90 (0.86–0.93)	0.94 (0.91–0.96)
	**JC**	1599 (639–5494)	250	93	659 (328–2981)	100 **	96	0.96 (0.95–0.97)	0.94 (0.91–0.95)
	**VZV**	1932 (489–4433)	250	87	718 (184–2033)	100 **	86	0.95 (0.93–0.97)	0.93 (0.90–0.95)

* Most measurements fall below the lower limit of quantitation of 100 MFI; ** a minimal cutoff of 100 MFI was applied to account for the lower limit of quantitation. Numerical correlation between the two data sets was assessed using Pearson’s r, while agreement is shown by the intraclass coefficient (ICC). Seroprev. = seroprevalence; IQR = interquartile range; MFI = median fluorescence intensity.

## Data Availability

Data are available on request from the corresponding author. They are not publicly available, due to data protection regulations.

## Supplementary Materials

The following supporting information can be downloaded at: https://www.mdpi.com/article/10.3390/microorganisms10071311/s1, Figure S1: Antibody responses to antigens from three endemic viruses measured in 142 paired serum and DBS samples and visualized as scatter plots; Figure S2: Antibody responses to eight non-structural and accessory antigens from SARS-CoV-2 measured in 142 paired serum and DBS samples and visualized as scatter plots.
