# Supplementary material for: Performance of Dried Blood Spot Samples in SARS-CoV-2 Serolomics
Source: Microorganisms. 2022 Jun 29;10(7):1311. doi: 10.3390/microorganisms10071311 (PMC9322257; doi:10.3390/microorganisms10071311)
Supplement: Supplementary file 1 [file microorganisms-10-01311-s001.zip › microorganisms-1731370-supplementary.pdf]

microorganisms

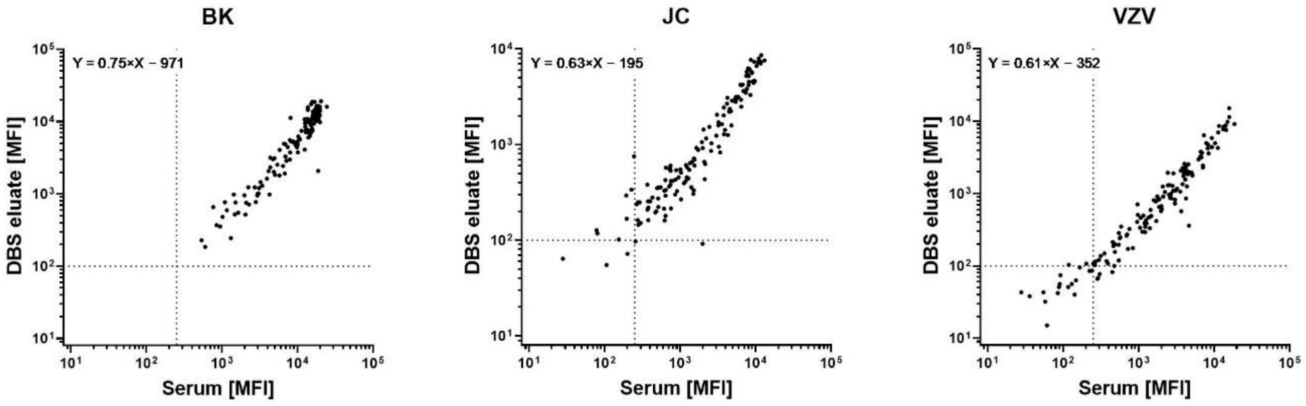

Supplementary Figure S1: *Antibody responses to antigens from three endemic viruses (VP1 of human Polyomaviruses BK and JC; gE/gI from varicella zoster virus, VZV) measured in 142 paired serum and DBS samples and visualized as scatter plots. Linear regression was applied to estimate the general trend between the two data sets.*

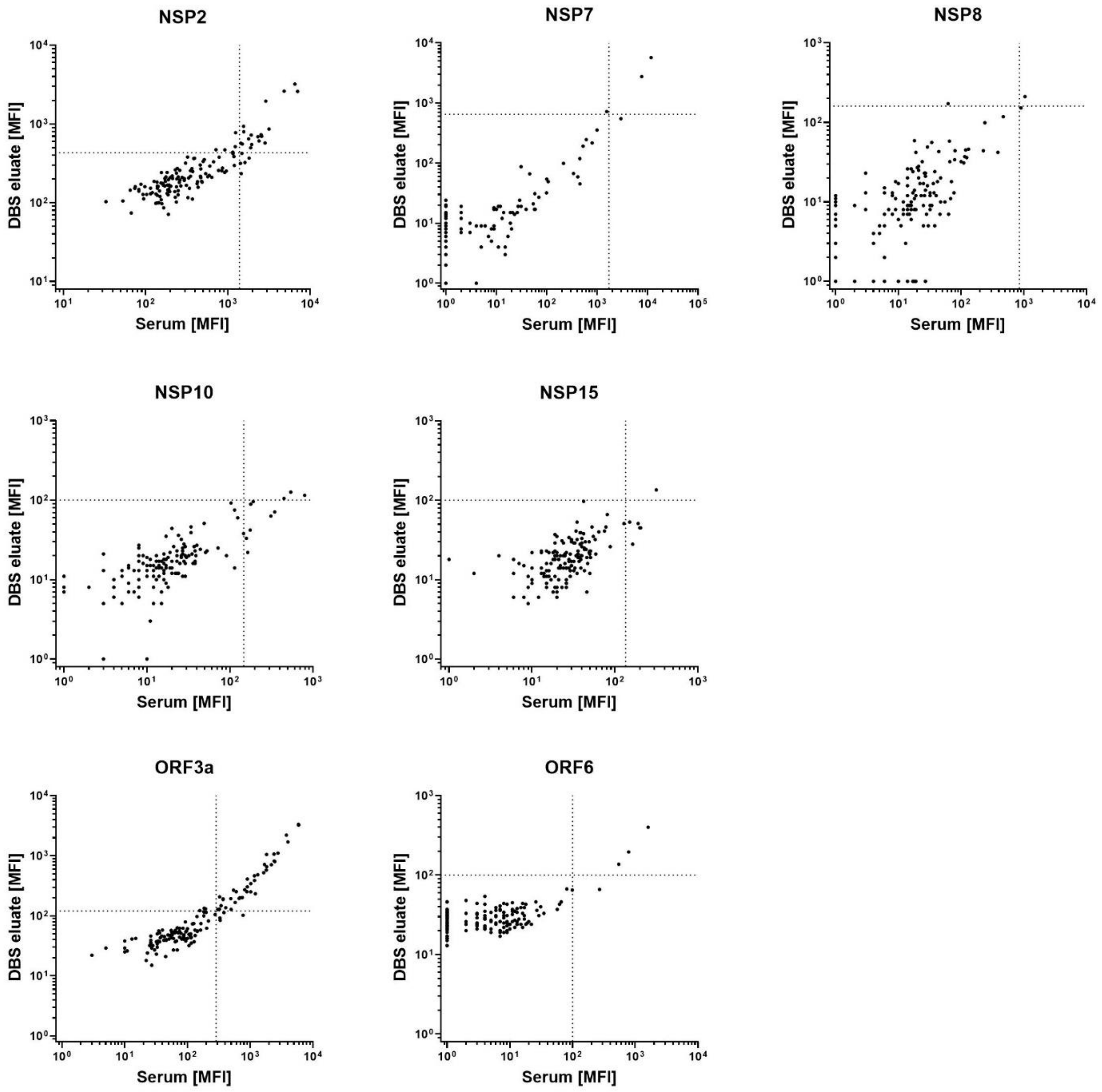

Supplementary Figure S2: *Antibody responses to eight non-structural and accessory antigens from SARS-CoV-2 measured in 142 paired serum and DBS samples and visualized as scatter plots. Measurements below the lower limit of quantification (100 MFI) show an elevated variance, potentially influenced by background effects, and are therefore not considered reliable. Different scales were chosen for each antigen according to respective seropositive signals.*
